# Co-Designing an Infant Early Childhood Mental Health Mobile App for Early Childhood Education Teachers' Professional Development: Community-Based Participatory Research Approach

**DOI:** 10.2196/66714

**Published:** 2025-06-02

**Authors:** Ruby Natale, Elizabeth Howe, Carolina Velasquez, Emperatiz Guzman Garcia, Karen Granja, Bianca Caceres, Elizabeth Erban, Tania Ramirez, Jason Jent

**Affiliations:** 1 School of Medicine/Mailman Center for Child Development University of Miami Miami, FL United States; 2 Community Health of South Florida Miami, FL United States; 3 Family Central Miami, FL United States; 4 Jewish Community Services Miami, FL United States

**Keywords:** early care and education, child care, social-emotional development, young children, professional development, infant early childhood mental health consultation, mobile apps, community-based participatory research, mixed methods, mobile phones

## Abstract

**Background:**

Many young children spend at least some time in early care and education programs, where they develop social-emotional skills that prepare them for future success. However, young children may exhibit behavioral challenges in these settings, negatively impacting their social-emotional development. It is critical that the early childhood workforce is prepared to support young children’s burgeoning social-emotional skills to address challenging behaviors in early care and education classrooms. Infant and early childhood mental health consultation is an evidence-informed approach for increasing teachers’ skills for managing young children’s emotions and behaviors. One mechanism to increase teachers’ access and use of the infant and early childhood mental health consultation programs is through on-demand mobile apps.

**Objective:**

This study aims to investigate 2 primary objectives: to document the development of the Jump Start on the Go (JS Go) app through community-based participatory research (CBPR) methodologies, and to evaluate and refine the app based on early childhood education (ECE) teacher feedback using a mixed methods assessment approach.

**Methods:**

This study used a community-based participatory research approach to design and evaluate the effectiveness of the JS Go app across 3 phases. In phase 1, a description of how the JS Go app was developed using CBPR principles is provided. In phase 2, teachers (n=12) were interviewed after reviewing mockups of the JS Go app to gather feedback about the interface and usefulness of the app to current and new teachers. Rapid qualitative analysis generated themes to inform phase 3 (n=31) of the study.

**Results:**

Phase 2 findings suggested that teachers viewed the app as aesthetically pleasing with concise information, but there were design and content features that needed to be refined to improve ease of use for accessing content. Teachers also described the app as beneficial and useful to both current and new ECE teachers and identified it as a tool to support sustainability for the use of JS practices. In phase 3, teachers rated the JS Go app favorably across all mHealth (mobile health) App Usability Questionnaire dimensions, including interface satisfaction (mean 6.12 on a 7-point scale), ease of use (mean 5.56), and usefulness (mean 5.37). Despite positive usability ratings, teachers expressed less certain intentions to adopt the app, scoring near the midpoint on the Technology Acceptance Model Instrument-Fast Form’s predicted future use scale (mean 1.60, –4 to +4-point scale). Implications for how the findings were used to make adaptions to the app are discussed. The next steps for testing the efficacy of the app in a randomized control trial are described.

**Conclusions:**

ECE teachers have overall positive perceptions about the value of the JS Go app. Future research will need to test the efficacy of the app for increasing and sustaining teacher’s use of JS practices.

## Introduction

### Background

Behavioral and mental health issues represent some of the greatest unaddressed challenges for young children, impacting their school readiness [1]. Preschool children who exhibit behavioral challenges are suspended and expelled from early care and education (ECE) programs at 3 times the rate of children in kindergarten-12th grade schools [2]. Moreover, racially and ethnically diverse children living in low-income communities are more likely to exhibit behavioral challenges leading to suspension or expulsion [1,2]. ECE programs play a vital role in promoting young children’s mental health by providing environments where children can develop social-emotional skills, including emotional regulation and resilience [3]. Research indicates that young children who attend high-quality ECE programs exhibit lower levels of behavioral problems and higher social competence compared to those who do not attend ECE programs [1,4].

ECE teachers in high-quality ECE programs can act as a protective factor by supporting young children’s burgeoning social-emotional development, thus preventing challenging behaviors [1,5]. Moreover, enhancing young children’s social-emotional development is linked with their school readiness, making a focus on this aspect of children’s development a mechanism for eliminating disparities [1]. However, there are limited professional development supports available to ECE teachers for implementing evidence-based social-emotional practices, particularly to support young children from low-income communities [6].

Infant early childhood mental health consultation (IECMHC) is an evidence-based model for supporting teachers in developing competencies for implementing social-emotional practices to support young children’s social competence and prevent challenging behaviors [7-9]. In an IECMHC model, teachers receive support from trained mental health consultants (MHC) to learn and practice new skills related to building social-emotional skills. Less than 10% of ECE teachers across the nation report access to MHC, yet over half report feeling that they struggle to manage children’s behavior [10]. IECMHC models offer accessible continuous professional development (CPD) for ECE teachers to guide the implementation of evidence-based social-emotional teaching practices. In a CPD model, teachers are responsible for creating their learning journey [11] which can increase their knowledge, sense of agency and confidence, perceptions of their competence, and ability to reflect on their practices leading to sustained use of new skills [12].

### Mobile App Technology

Mobile technology is a CPD mechanism that may increase the uptake of IECMHC practices. The prevalence of mobile technology is increasing, suggesting that mobile apps may be a critical mechanism for eliminating disparities in service receipt. For example, 258 billion apps were downloaded in 2022 [13], with the World Development Report indicating that the poorest households are more likely to have a smartphone than electricity [14], and Hispanic adults are one of the leading users of smartphones [15].

Mobile apps are critical CPD tools for just-in-time, on-demand learning [16]. Mobile apps address the personal agency of the learner to decide when, where, and how they want to learn [16]. Mobile apps are becoming an increasingly common mechanism for providing CPD support in ECE programs [17]. ECE teachers have made progress in integrating digital tools and technology into their everyday practice [18] and these tools can support the sustainability of new skills by teachers [18]. In the ECE space, mobile apps are used for a variety of pedagogical purposes, including teaching strategies to increase young children’s physical activity, implement educational activities (eg, science, technology, engineering, and mathematics), and increase teachers’ creative competence [19-21].

There is emerging evidence that mobile health (mHealth) apps can support the development of a therapeutic alliance between an MHC and teacher [22] which is a key component of IECMHC models. However, there is limited research on how mobile apps support teachers in building relationships with children and collaborating with families [23], which are also essential elements of an IECMHC model. To our knowledge, there is yet to be a mobile app dedicated to providing IECMHC content to ECE teachers.

### The Study

To address a gap in IECMHC mobile technology, our team engaged in a community-based participatory research (CBPR) model to co-develop a mental health app to provide the Jump Start (JS) program, an IECMHC model [7]. Jump Start on the Go (JS Go) is a mechanism for providing CPD to ECE teachers. The first objective of this study was to document the development and features of the JS Go mobile app, which emerged through CBPR methodologies The second objective of this investigation was to systematically evaluate and refine the JS Go app through comprehensive feedback from ECE teachers, using a mixed methods approach to assess both implementation successes and opportunities for app enhancement. The findings were used to evaluate teachers’ perceptions of the perceived ease of use, usefulness, user interface, and the predicted future use of the JS Go mobile app as a CPD tool for the delivery of IECMHC practices to ECE teachers in Miami-Dade and Broward Counties, Florida. Children and teachers in ECE programs in Miami-Dade and Broward Counties are racially and ethnically diverse, and many live in lower-income communities with multiple neighborhood risk factors contributing to poorer school outcomes. Given the population of children, teachers, and ECE programs in these counties, it is a priority that the community voice and perspective contribute to the development and evaluation of JS Go.

## Methods

### Study Design

The study was implemented in three phases using exploratory sequential mixed methods. Phase 1 focused on the use of CBPR principles to develop the app. Phase 2 was dedicated to preliminary user testing using qualitative interviews with a small number of ECE teachers who reviewed mock-ups of JS Go to answer the following research questions in order to refine the app: (1) What feedback do ECE teachers have about the interface and aesthetics of the JS Go app for ease of use? (2) How do ECE teachers describe the usefulness of the JS Go app for both current and new teachers? Phase 3 used formal real-world testing of the app by ECE teachers currently receiving JS IECMHC services to answer the following questions: (1) What are ECE teachers ratings for the ease of use, their satisfaction, the user interface, and the usefulness of JS Go for accessing IECMHC content using JS Go? (2) What are teachers’ ratings for the usefulness, ease of use, and predicted future use of the JS Go app as a mechanism for consultation?

### Ethical Considerations

This study was approved by the University of Miami School of Medicine, Human Research Subjects Internal Review Board (approval number 20231026). Written informed consent was obtained from all participants prior to data collection. Study data are anonymous, and all participant responses were deidentified using unique identification numbers. Data were stored in cloud-based electronic systems for security. Participants in phase 2 received a US $20 gift card for the interview and participants in phase 3 received a US $20 gift card for beta testing the app and a certificate for 5 hours of in-service training for their participation in the study.

### Study Phases

#### Phase 1: CBPR for Mobile App Development

CBPR is a collaborative approach to research that actively involves community members in the research process to address issues of local concern and promote social change [24]. We applied a CBPR framework developed by Israel et al [25,26] that includes 9 principles (Textbox 1). Unlike traditional research methods that often distance researchers from the communities they study, CBPR seeks to equalize the research process by engaging community members as equal partners in the design, implementation, and evaluation of research projects [25,26]. The CBPR approach emphasizes mutual respect, shared decision-making, and a focus on actionable outcomes that benefit the community [27]. CBPR has been shown to enhance the relevance and quality of research by ensuring that it reflects the community's needs and perspectives, thus improving the likelihood of successful interventions and sustainable solutions [28].

**Description of how Israel et al’s 9 principles of community-based participatory research were used to develop and test Jump Start on the Go (JS Go).**

Through our long history of effective community partnerships, we address the following 9 principles of CBPR.

• Recognizing the community as a unit of identity, we acknowledge our partners’ unique and individual contributions to ensure the cultural appropriateness and accessibility of JS on the Go.

• Building on community strengths and resources by including the community voice in the design, development, and refinement of JS on the Go.

• Facilitating collaborative equitable partnerships in all phases of the research by including our partners in all stages of JS on the Go for co-development, refinement, testing, and dissemination.

• Involving an empowering and power-sharing process that attends to social inequities by including our community partners who are trusted sources as co-investigators to foster co-learning and ensure reach to early childhood education (ECE) programs in neighborhoods’ historic disparities.

• Integrating and achieving a balance between knowledge generation and intervention for the mutual benefit of all partners through quarterly co-creation meetings with our community partners to design, develop, and refine JS on the Go.

• Focusing on the local relevance of public health problems and ecological perspectives that recognize and attend to multiple determinants of health by including partners from communities with historic disparities in access to ECE programs resulting in disproportionate outcomes to obtain their perspectives and gain an understanding of their backgrounds.

• Involving systems development using a cyclical iterative process through our exploratory sequential mixed methods research design implemented in multiple phases to gain an understanding of the acceptability, feasibility, usefulness, and usability of JS on the Go.

• Disseminating findings and knowledge gained to all partners and involving them in the dissemination process by co-developing publications and presentations about JS on the Go.

• Involving a long-term process and commitment to sustainability with pledged support from our community partners to support the development of future grants to bring JS to Go to scale across Miami-Dade County and nationwide.

Using a CBPR approach for the development and testing of a mobile app can enhance its relevance, usability, and effectiveness by incorporating the perspectives of community members throughout the process [15,29,30]. This approach can ensure that the mobile app meets the specific needs of the intended users and fosters community engagement and ownership [30]. A CBPR approach for mobile app development and testing is imperative for use in low-income, racially, and ethnically diverse communities [15,29] because it allows community members to tailor technology to meet their unique and individualized needs using a strengths-based approach, increasing the likelihood of their sustained use of the tool [30].

The app was developed using the 9 principles of CBPR by creating a community advisory committee. Our partners in the community advisory committee are composed of 3 community-based organizations: Jewish Community Services, Community Health of South Florida, and Family Central. All of these community-based organizations provide JS MHC in ECE programs in Miami-Dade County. The community advisory committee also included members of the Women’s Fund of Miami-Dade County and their grantees. The Women’s Fund is a nonprofit advocacy organization investing in initiatives for girls and women, including the early childhood workforce, in Miami-Dade County.

The community advisory committee oversaw the development of the app using the 8 dimensions of the Ecological Validity Model (Textbox 2 [31]) to ensure that the app had the necessary cultural and linguistic adaptations to reduce health disparities [31,32]. The Ecological Validity Model is the most widely used approach for culturally adapting interventions to ensure they are usable for the intended audience and feasible to use within the desired settings [33]. Our community advisory committee assisted with co-developing scripts for the videos and modifying content to ensure their relevance to ECE programs within their communities. Given that the app was multilingual, the community advisory committee ensured that the dialect of Spanish and Haitian Creole that was used in videos and text within the app aligned with local community linguistic patterns. Agendas during the initial meetings included explanations of the purpose and goals of the study, describing the JS IECMHC intervention, sharing prototypes, and eliciting feedback. We revised the multilevel intervention based on community advisory committee feedback, presenting the revised content of JS Go at subsequent meetings. If there were extensive changes based on feedback during the final meetings, we requested their permission to have additional meetings to ensure that all feedback was appropriately incorporated.

**The 8 dimensions of the Ecological Validity Model used in the development of Jump Start on the Go (JS Go) and the methods for testing.**

- Language: Culturally appropriate language
- Persons: Role of ethnic or racial similarities and differences between client and therapist in shaping the relationship
- Metaphors: Symbols and concepts shared by populations
- Content: Cultural knowledge, values, traditions—the uniqueness of groups
- Concepts: Concepts consonant with culture and context
- Goals: Transmission of positive and adaptive cultural values from the culture of origin
- Methods: Development and cultural adaptation of treatment methods
- Context: Consideration of changing contexts in assessment during treatment or intervention

#### Phase 2: Preliminary Qualitative End User Testing

##### Participants and Procedures

After the content of the JS Go app was developed, we interviewed 12 teachers between April 2024 and May 2024 on the usability and feasibility of JS Go. We purposefully recruited participants (n=12) from 12 ECE centers in low-income tracts within Miami-Dade and Broward Counties currently participating in the JS program. Participants were selected based on their role as program champions in the larger randomized control trial for JS and their participation in supplemental professional development to obtain their infant mental health endorsement. There was a total of 32 program champions who completed the professional development. Participants were notified about the study by their MHC. Once participants were notified, they were contacted via email by the interviewer to schedule the interview.

##### Measures

The data from phase 1 was collected as part of a larger qualitative study. Interviewers used a semistructured interview guide developed by the research team for data collection. The questions in the interview guide were developed using the Reach, Effectiveness, Adoption, Implementation, and Maintenance framework [34,35]. The Reach, Effectiveness, Adoption, Implementation, and Maintenance framework uses an implementation science lens for evaluating interventions to determine their applicability to real-world settings (Textbox 3 and Multimedia Appendix 1). The questions posed to participants in phase one focused on the maintenance of JS Go.

**Preliminary end user testing interview questions for phase 2.**

Phase 2: Interview questions

- **What feedback do you have regarding the 5 screenshots?**
  Home page
  Journey
  Providers
  Services
  Videos
- What additional features or changes would you suggest for the app?
- How helpful can this app be for helping teachers sustain their use of Jump Start (JS) practices?
- How helpful can this app be for helping new teachers learn to use JS practices?
- Are there any other comments, questions, or concerns you would like to share about the app?

Interviews with teachers were conducted by a Master level MHC unfamiliar to the teacher to minimize bias. Interviews were conducted one-on-one with participants using Zoom videoconferencing software (Zoom Video Communications). Interviews were conducted in either English or Spanish depending on the preference of the participant. The average length of the interviews was 45 minutes 32 seconds with a range of 37 minutes 11 seconds to 65 minutes 29 seconds.

Formal user testing was captured via think-aloud walkthrough interviews. Think-aloud walkthroughs are a common method to evaluate the usability of apps and involve users verbalizing experiences and perceptions as they navigate through the app [36-39]. Participants are able to provide both positive and negative reactions to their experiences during the walkthrough to minimize bias. Mock-ups of the app were shared with teachers via screenshots of each page by the MHC (Multimedia Appendix 1). This allowed participants to react to the features of the app including the aesthetics and content. Transcripts of the recorded interviews were downloaded from Zoom to a secure cloud storage platform and deidentified. Transcripts were cleaned by the lead interviewer and translated, then back-translated by certified translators prior to analysis.

#### Phase 3: Formal Quantitative Real-World Testing

##### Participants and Procedures

Following the completion of phase 2, and consistent with CBPR, we then engaged in a 3-month feasibility field study (June 2024-August 2024) in which MHC from our 3 community-based organizations co-facilitated formal real-world, or beta testing, of the JS Go app with teachers. Teachers in phase 3 did not participate in the interviews during phase 2. Purposeful sampling was used to have MHC identify ECE teachers they were currently or had recently worked with to participate in phase 3 of the study (n=31). ECE teachers came from 18 centers in low-income tracts in Miami-Dade and Broward Counties in South Florida participating in the JS program. Participants were eligible to participate if they were currently or recently enrolled (within 3 months) in the JS program. Participants were notified by their consultant to determine their interest and informed consent was obtained prior to beta testing.

Formal real-world testing occurred over a 3-week period during regularly scheduled consultation sessions between the teacher and their MHC. During consultations, MHC used the app in tandem with the teachers. Teachers completed surveys following beta testing with the app. Surveys were completed by teachers through the JS Go app. MHC did not have access to teachers’ responses to minimize bias. Teachers’ responses to the surveys in the app were linked to a secure cloud-based data management platform, REDCap (Research Electronic Data Capture, Vanderbilt University) [40,41].

Formal real-world testing was completed via rapid-cycle prototyping in 3 cycles with each cycle lasting 3 weeks. In cycle 1, seven centers and 16 teachers participated; in cycle 2, seven centers and 13 teachers participated; and in cycle 3, two centers and 4 teachers participated. During this time period, teachers used the app as part of their consultation sessions with their MHC. Teachers completed surveys after using the app during their consultations to gauge their perceptions of the acceptability, feasibility, and usability of the app’s features and the functionality of the app for telehealth consultation. To aid in using the survey data open-ended questions were used during weekly meetings between the app developer and MHC to address poorly scoring items. Examples of issues addressed between cycles included how teachers create their profile and log in to the app, ensuring resources are downloaded to their smartphone rather than opening in a new browser window, and ensuring infographics are available in all languages.

Following the completion of each cycle, we made iterative changes to the app based on the survey data and feedback from MHC.

##### Measures

During phase 3, teachers completed the mHealth App Usability Questionnaire (MAUQ) in English and Spanish [42]. The mHealth App Usability Questionnaire is a short reliable and customizable questionnaire to assess the usability of mobile apps to gauge teachers’ perceptions of the usefulness of JS Go and its acceptability as a tool for delivering IECMHC. The mHealth App Usability Questionnaire has 18 items organized into 3 subscales; ease of use and satisfaction, system information arrangement, and usefulness. Participants rated JS Go using a 7-point Likert scale (1=strongly disagree to 7=strongly agree). The internal consistency for all scales within the mHealth App Usability Questionnaire measure was high ranging from α=.876 (ease of use and satisfaction) and α=.908 (system information arrangement) to α=.843 (usefulness) [42].

In addition to the MAUQ, the Technology Acceptance Model Instrument-Fast Form (FF-TAM) in English and Spanish was used to measure teachers’ beliefs about the functionality of the JS Go app as a mechanism for teleconsultation [43]. The FF-TAM measures teachers’ acceptance of technology which predicts their perceptions of the ease of use of the app as a telehealth consultation mechanism. The FF-TAM has 16 items organized into 3 subscales; usefulness, ease of use, and predicted future use. The FF_TAM evaluates attitudes toward technology use and can be modified to include the technology of interest. In this study, the form was adapted to measure participants’ beliefs about the JS Go app for teleconsultation. Participants rate their beliefs about the JS Go app using an 8-point semantic differential scale ranging from –4=most negative rating of an item to +4=most positive rating of an item. The Cronbach α for the scales ranged from 0.935 to 0.957 [43].

### Data Analysis

During phase 2, rapid qualitative analysis was used to identify pertinent themes from the interviews with teachers [44-48]. The rapid qualitative analysis allowed the software developer to make real-time changes to the app based on feedback from teachers in phase 2 to optimize beta testing in phase 3. Rapid qualitative analysis has standards for rigor [47] and the use of this type of analysis is supported by implementation science research. Rapid qualitative analysis is a method of expediting qualitative analysis to address pressing issues, such as the timely adaptation of the app to meet the needs of the ECE workforce. The process involves summarizing interview transcripts using a standard template and subsequently transferring key points into a matrix to explore relevant themes [48]. The MHC who conducted the interview completed the initial summaries. A second member of our research team transferred the key points from the interview summary into the matrix. A third member of the team used the matrix to identify themes, which were then refined through discussion with study investigators and other members of the team.

Quantitative data collected during phase 3 were analyzed using SPSS (IBM Corp) [49]. The Cronbach α was calculated for both measures, including each subscale. Descriptive statistics were used to calculate the means and SDs for each of the subscales for both the MAUQ and FF-TAM as well as the total score and item range.

## Results

### JS Go

The content of JS Go is based on the evidence-based JS program [50]. JS includes 4 pillars based on Caring for Our Children–National Health and Safety Standards [51]. The 4 pillars are safety and planning, trauma-informed behavior support, adult self-care, and effective communication. JS Go encourages independent learning by allowing users to drive their access to the app by navigating content based on their personal needs. The app includes a self-assessment for using JS practices, infographics describing JS content, videos illustrating JS practices in action, and access to other supplemental materials and resources. JS Go also includes an artificial intelligence–driven chatbot that is specifically trained on JS content. Teacher users can ask questions and receive responses based on machine learning. An app, such as JS Go, developed with community partners via CBPR, could serve as a scalable paradigm shift by bringing MHC to teachers who need it most in real time.

### Participant Characteristics

All of the teachers in phase 2 were female. Most of the teachers reported they were White and Hispanic, and their primary language was Spanish. The majority of the teachers were between the ages of 40 and 49 years and most have a bachelor’s degree (Table 1).

All of the teachers in phase 3 were female. Most of the teachers reported they were White and Hispanic, and their primary language was Spanish. The majority of the teachers were between the ages of 30 and 39 years and most reported having some college, which includes technical training and holding an associate’s degree (Table 1).

**Table 1:** Participant characteristics for preliminary end user testing (phase 2) and formal real-world testing (phase 3) of JS Go.

Characteristics			Phase 2 participants		Phase 3 participants
**Sex, n (%)^a^**					
	Female	12 (100)		31 (100)	
	Male	0 (0)		0 (0)	
**Age (years), n (%)^b^**					
	20-29	1 (9)		1 (3)	
	30-39	2 (18)		10 (32)	
	40-49	6 (55)		7 (23)	
	50-59	1 (9)		8 (26)	
	More than 60	1 (9)		5 (16)	
**Education level, n (%)^c^**					
	High school or GED^d^	2 (18)		4 (15)	
	Some college^e^	3 (27)		13 (48)	
	Bachelors	4 (36)		7 (23)	
	Graduate	2 (18)		4 (15)	
**Primary language, n (%)^a^**					
	English	3 (25)		7 (23)	
	Spanish	9 (75)		24 (77)	
**Race, n (%)^a^**					
	Black non-Hispanic	1 (8)		5 (16)	
	White	11 (92)		26 (84)	
**Ethnicity, n (%)^a^**					
	Hispanic	11 (92)		27 (87)	
	Non-Hispanic	1 (8)		4 (13)	

^a^12 participants in phase 2 and 31 in phase 3.

^b^11 participants in phase 2 and 31 in phase 3.

^c^11 participants in phase 2 and 27 in phase 3.

^d^GED: General Educational Development.

^e^Some college includes technical training and associate's degree programs.

### Phase 2: Preliminary Qualitative End User Testing

#### Research Questions 1. JS Go Interface

**Figure 1:**
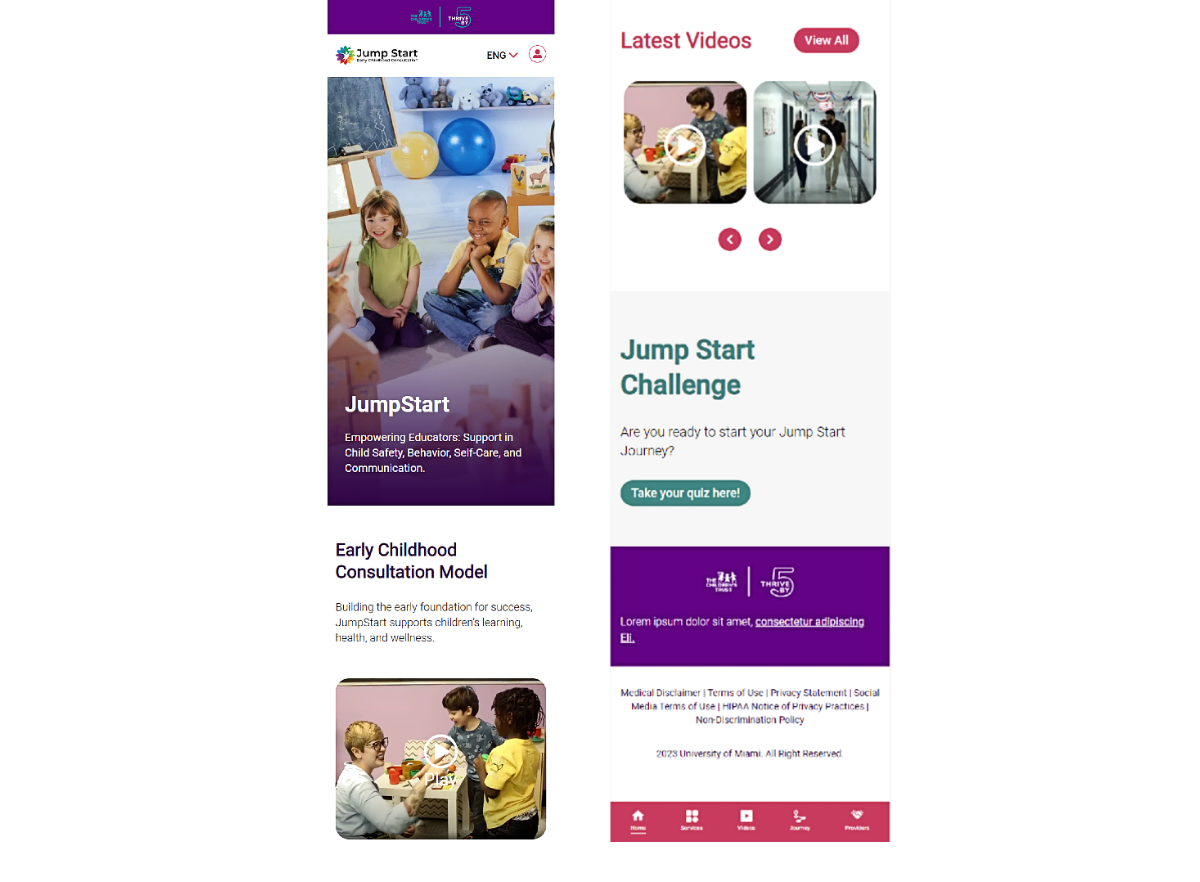
JS Go (Jump Start on the Go) home page.

Two themes were generated regarding teachers’ perception of the JS Go interface for ease of use. Figure 1 shows the JS Go home page The first theme was the high perceived ease of use with 3 subthemes: concise information, aesthetically pleasing, and language accessibility. Most of the participants reported they were satisfied with the JS Go app’s appearance as it allowed for ease of use. One participant noted, “It’s, it’s been very good, I like that. You know, the first thing we get is a little introduction; it’s quite good.” The participants also described the JS Go app’s interface as clear and concise, with the training section as straightforward and direct. For example, 1 respondent stated “The good thing about the page is that I see it with little information. Pages loaded with information, what they tend to do is confuse us.” High perceived ease of use also included the appearance of the content for different sections of the app and for resources such as videos. Participants described the aesthetics as “eye-catching” with a “minimalistic approach” that is “straightforward.” Participants also appreciated the translation feature and language change option. One respondent stated the following.

For me, it’s fine. And you even have it translated. Because what it says up there that says SPA (Spanish), it’s very important that it’s translated. There are many teachers who don’t speak English

Teacher #3, female

Another participant noted the following.

It’s a well-thought-out app, but the only thing I see is that it has an option to change languages. If it has an option for Spanish, it would be good to create one for Creole as well because, at least in the area where we work, there are many teachers who are Haitian. They do speak English, but obviously, their first language is Creole

Teacher #2, female

The second theme generated included suggestions related to the app’s visual attractiveness, functionality, and app content (Figure 1). Suggestions related to aesthetics included color choices, and reducing white space to optimize visibility on smartphones. Suggestions related to functionality include adding a search bar to the home page, changing the wording on the home page to “empowering educators and families,” and improving the navigation of the home page to make it clearer. Other suggestions related to functionality include having a space for specific child cases to obtain follow-up strategies akin to an individual behavior plan and how the app is structured, such as including a start button. Suggestions about the content included requests for additional education videos that are concise and directly related to the JS program and videos demonstrating how to resolve various classroom situations. While yet another suggestion from a participant regarding content was to integrate songs as supplementary tools for classroom use.

#### Research Question 2. Helpfulness of JS Go

There were 4 themes generated describing how ECE teachers and directors perceive the helpfulness of the JS Go app; benefits, usefulness for current teachers, usefulness for new teachers, and sustainability (Figure 1). Participants describe the JS Go app as beneficial for accessing resources such as handouts, videos, courses, and infographics to facilitate teachers’ jobs for timely receipt of information. Benefits also included the use of the app for planning and access to additional support when an IECMH consultant is not available. For example, 1 respondent noted the following.

I see it as an extra resource, right at their fingertips. That they can even like on their break, can look something up. I mean, I know I rather look up things on the phone or like an app, then go to like a website like sometimes I will be doing something on my phone. And I'm in front of the computer at work. But just because it was so much easier to just click, click, and just keep doing it on the phone

Teacher #3, female

Participants perceived the JS Go app as useful for current teachers for reviewing, refreshing, or reinforcing the use of JS practices for on-demand learning. For example, 1 respondent stated the following.

I think so because when you have doubts about something, you can go there and search the application. I think so. There are times when you have doubts about something or you don't remember something and you say 'you have to do this, I have the doubt' and you go in and you can reflect and look and do...

Teacher #1, female

Participants perceived the JS Go app as especially important for new teachers, especially following the workforce shortage resulting from the pandemic [52]. Participants described the app as useful for introducing JS content and having access to direct resources. The benefits included increasing new teachers’ autonomy, bridging the gap between theory and practice, and having direct access to resources when things are not working in the classroom. For example, 1 participant stated, “New teachers have two ways of learning, either through theory when they go to school or during training, but often it doesn’t apply in practice.” Another respondent noted the following.

For her, it would be like a learning method, telling her, 'Look, here you have this tool that will help you when you feel things aren't working, when you feel frustrated, or when you have any problem. Open the app and, just like you study the courses or anything else, start studying the app

Teacher #7, female

Participants described the JS Go app as important for promoting the use of JS practices by providing supportive resources such as videos and classroom management strategies. The potential of the app for sustainability can be attributed to the practicality and clarity of the videos, and by providing an immediate resource to address unexpected learning situations, which is especially important when the director or consultant is not available. For example, 1 respondent noted the following.

The videos, the videos then stay a lot because that is like a practical example that was given and sometimes one looks for videos and sometimes they are difficult to find because they are like inside a link and the link that I send you something else and then that it has this direct and that you can see it. With the titles and names I think it's excellent

Teacher #4, female

The themes generated using rapid qualitative analysis are enlisted in Textbox 4.

**Relevant themes generated through rapid qualitative analysis.**

**Research question 1: Teachers’ perceptions for the interface of Jump Start on the Go (JS Go)**

1. High perceived use
  - Concise information
  - Aesthetically pleasing
  - Language accessibility
2. Suggestions
  - Aesthetics
  - Functionality
  - Content

**Research question 2: Teachers’ perceptions for the helpfulness of JS Go**

1. Benefits
  Access to resources
  Planning
  Access to support
2. Usefulness for current teachers
  On-demand learning
3. Usefulness for new teachers
  Content introduction
  Access to resources
  Autonomy
4. Sustainability
  Supportive resources
  Practicality
  Immediate resource

### Phase 3: Formal Quantitative Real-World Testing

#### mHealth App Usability Questionnaire

The total mean for the measure was 5.64 with an SD of 1.71, indicating participants were satisfied with the JS Go app and believed it is easy to use and useful (Table 2). The mean subscale scores across all 3 cycles of testing indicated that teachers had a positive perception of the ease of use, satisfaction with the app interface, and usefulness of the JS Go app.

**Table 2:** Teacher perceptions of the ease of use, satisfaction, usefulness, and predicted future use of the JS Go app (N=31).

Scale			Mean (SD)		Item response range (lowest to highest)
**mHealth App Usability Questionnaire^a^**					
	Ease of use	5.56 (1.48)		1.80-7.00	
	Interface and satisfaction	6.12 (1.02)		3.29-7.00	
	Usefulness	5.37 (1.38)		1.83-7.00	
**Technology Acceptance Model Instrument-Fast Form^b^**					
	Usefulness	2.97 (1.56)		–2.00 to 4.00	
	Ease of use	2.76 (1.77)		–2.67 to 4.00	
	Predicted future use	1.60 (2.94)		–4.00 to 4.00	

^a^mHealth: mobile health; mHealth App Usability Questionnaire scores range from 1=most negative rating to 7=most positive rating.

^b^Technology Acceptance Model Instrument-Fast Form scores range from –4=most negative rating to +4=most positive rating.

#### Technology Acceptance Model Instrument-Fast Form

The total mean for the measure was 2.44 with an SD of 0.52. The mean subscale scores across all 3 cycles of testing indicate that the teachers perceived JS Go to be easy to use and valuable but they had less favorable beliefs about using the app in the future (Table 2).

## Discussion

### Principal Findings

This study provides preliminary evidence regarding the benefits and usefulness of the JS Go mobile app as a CPD tool for teachers in accessing IECMHC practices in a timely, on-demand manner. The ECE teachers in this sample were all from centers in low-income tracts in Miami-Dade and Broward Counties, Florida. Focusing on the delivery of CPD in these centers is critical for minimizing disparities typically encountered by young children in these underserved neighborhoods [53]. To further address the diversity of the communities and where these centers reside to ensure the app’s social validity, we used a CBPR approach based on 9 principles to develop and test JS Go [54].

By using a CBPR approach, we recognized and valued our community partners and the neighborhoods they represent, as important units of identity. Recognizing each community as a unit of analysis allows us to build on their strengths and resources by facilitating collaborative and equitable partnerships in all phases of our research. Through our ongoing and regular meetings with our community partners, we achieved a balance between knowledge generation and intervention about JS Go, mutually benefitting all partners. Including our community partners as co-investigators affirmed their value as trusted knowledgeable sources assuring our reach to ECE teachers and programs in their neighborhoods to understand their experiences with multiple determinants of health.

Similar to other CBPR research, we used an iterative approach to develop, refine, and test the JS Go app across multiple phases [15,29,30]. One aspect of the JS Go that appears to be particularly appealing to ECE teachers’ is the access the app provides to IECMHC resources, which is in line with prior research on mobile technology using a CBPR approach [30]. One reason for this may be due to support for not only implementing IECMHC practices but also for successfully navigating the app itself as a CPD tool [30]. A strength of our research design and approach is the use of ECE teachers in the communities we strive to reach as end users to obtain valid and culturally relevant information about the usability of the app [30].

This study used an exploratory sequential mixed methods design to gather information from ECE teachers working in existing centers participating in the JS program to inform app development and evaluation across 3 phases. The iterative design allowed us to include our community partners’ voices and perspectives during each phase to ensure the acceptability, feasibility, and usefulness of JS Go for ECE teachers and programs in underserved neighborhoods. Finally, we are ensuring a long-term commitment to sustainability, with pledged support from our community partners, who will support ongoing dissemination about JS Go as a germane CPD tool. This long-term commitment can lead to widespread use of JS Go across South Florida and nationwide.

Notably, the ECE teachers who participated in this study were from ECE programs in typically underresourced neighborhoods serving diverse children. To address the diversity of teachers and ECE programs in these neighborhoods, we used the Ecological Validity Model as a framework to guide the development and evaluation of JS Go, ensuring it is culturally relevant and generalizable to the communities in Miami-Dade and Broward Counties [31,32]. Given our attention to the cultural contexts of the neighborhoods in which JS Go will be implemented, it is culturally sensitive. For example, the teachers can use the app in any of three languages, English, Spanish, and Haitian Creole, with teacher participants indicating their appreciation for the availability of app content in multiple languages.

### Phases 2 and 3

ECE teachers had overall favorable perceptions of the JS Go mobile app, including their satisfaction and high perceived use of the app’s interface. Feedback garnered from teachers about the app’s interface during the qualitative interviews in phase 2 and quantitative real-world testing was shared with the app developer to aid in making adjustments to improve functionality. Users found the app to be easy to use, functional, beneficial, and useful for both current and new ECE teachers. Yet, they were less likely to rate it as a tool they would use in the future for telehealth consultation, suggesting some hesitancy with mobile apps as useful for this form of professional development. This was in contrast to their favorable report about using the app where they indicated they would use the app again in the future. One explanation for this finding could be that the teachers in this study were older, and research suggests that the age of end user influences their willingness to use mobile technology as a professional development tool while still valuing access to IECMHC content via an app [55].

A CPD tool that delivers individualized IECMHC content in a timely, on-demand manner can be a critical mechanism for training new ECE teachers while addressing their personal agency, confidence, and competence in their new role [12]. Addressing new teacher training, agency, confidence, and competence is vital, given the ongoing ECE workforce crisis exacerbated by the COVID-19 pandemic [52]. Thus, JS Go has the potential to be a viable tool for onboarding new teachers by giving them immediate access to the app with essential information on how to improve classroom quality. Additionally, teachers felt JS Go is an important mechanism to support the sustainability of JS practices by ECE teachers following receipt of IECMH consultation. Implementation science cites the sustainability of evidence-based practices as critical for scaling interventions [56]. The app would allow us to sustain and quadruple our reach across South Florida. As such, the JS Go app has the potential to increase equitable access for teachers to evidence-based IECMHC content helping to eliminate disparities typically experienced by young children in these communities. The JS Go app is a tool that could offer a paradigm shift in how teachers receive timely on-demand CPD tailored to their individual learning needs, increasing their autonomy and potentially leading to sustained use of JS practices.

This study contributes to a growing body of research regarding the viability of mobile apps for the delivery of CPD [19-21,57]. Research indicates ECE teachers integrate digital tools into their everyday practice to construct their learning using informal resources [17,18]. Mobile apps offer a tool for timely, on-demand learning, which can be a critical feature for ECE teachers working with diverse children in dynamic classrooms [16,18]. Additionally, mobile apps are a viable option for access to help and resources when in-person services are unavailable [58]. Mobile apps are also a CPD tool with the potential to support teachers’ sustained use of new practices [57], critical for scaling evidence-based interventions [58]. Prior research shows that mobile apps are rated highly for their usability [59], which is in line with the findings in this study. However, there is a paucity of research on the use of mobile apps to support young children’s social-emotional development, such as building relationships and communicating with families [22,23].

### Next Steps

Future research by our team will pilot the efficacy of the JS Go app through a randomized controlled trial in six centers in South Florida. Each of the centers will be randomly assigned (via a random number table) to 1 of 2 arms, the intervention arm or the attention control arm. Both arms will be followed for 1 school year and then a 1-month follow-up. Centers in the JS Go arm will receive their IECMHC through the app in which they will participate in 12-24 mental health consultation sessions. Centers in the attention control arm will receive business-as-usual JS mental health consultation. We will implement the randomized controlled trial using CBPR using the 9 principles of Israel et al [54]. Our community advisory committee will continue to meet quarterly to review findings and recommendations and to support dissemination and sustainability. Meeting with partners will allow us to achieve knowledge generation that will mutually benefit the community and the research team by focusing on the local relevant public health issue regarding implementing IECMHC to support young children’s social-emotional development and school readiness. Involving community partners in the research process will also allow us to build on each community's unique strengths and resources to attend to the social inequities ECE teachers and young children experience in these neighborhoods.

### Limitations

There are several limitations to this study that should be noted. First, participants in qualitative interviews and quantitative testing were not identical, although both came from the same population of teachers currently participating in the JS program. Different populations pose a threat to the legitimation of making inferences from both qualitative and quantitative data [60,61]. However, using this sampling strategy is in line with the purpose of this research by allowing us to compare perceptions about the app from 2 separate groups of teachers from the same population. Additionally, the sample for the quantitative testing was small, limiting generalizability about the satisfaction and usefulness of the app with teachers who are not participating in the JS program, thus it is not possible to know the perceptions of these teachers [60]. Moreover, purposeful sampling of teachers may have introduced bias as MHC selected teachers based on their perceptions about teachers’ ability to engage in the research. We attempted to minimize bias throughout the study in our methodology. However, it is possible that the response of teachers participating in phase 3 may not be entirely independent given their close relationship with their MHC. Finally given the different sample sizes of teachers in each cycle, with decreasing sample sizes, we elected to present total scores for the overall sample. The changes in scores across the 3 cycles are difficult to interpret given the differences in the sample sizes of teachers.

### Conclusions

This paper describes the use of CBPR to develop the JS Go mobile app, a tool for delivering IECMHC content to ECE teachers in real time. The communities in this study face ongoing disparities in access to high-quality ECE service. Community stakeholders provided input at all stages of the app’s development, including participation in data collection, allowing for contextualization of the data. This study used sequential exploratory mixed methods to evaluate the usability, acceptability, and feasibility of JS Go. Exploratory sequential mixed methods research is particularly salient for evaluating new approaches, as it allows for nuanced feedback via qualitative methods, and measurable input through quantitative data [60,61]. Specific to our population, the JS Go pilot data shows teacher satisfaction, given quick, easy, anytime-anywhere access to IECMHC information. To our knowledge, the JS Go app is the first mobile app that addresses the CPD needs of ECE teachers in implementing IECMHC content.

## Appendix

Multimedia Appendix 1 Mockups of JS Go (Jump Start on the Go) app.

## Abbreviations

CBPR: community-based participatory research
CPD: continuous professional development
ECE: early care and education
FF-TAM: Technology Acceptance Model Instrument-Fast Form
IECMHC: infant and early childhood mental health consultation
JS: Jump Start
JS Go: Jump Start on the Go
MAUQ: mHealth App Usability Questionnaire
MHC: mental health consultant
mHealth: mobile health
REDCap: Research Electronic Data Capture

## Data Availability

The datasets generated and analyzed during this study are available from the corresponding author on reasonable request.
