# Supplementary material for: Co-Designing an Infant Early Childhood Mental Health Mobile App for Early Childhood Education Teachers' Professional Development: Community-Based Participatory Research Approach
Source: JMIR Form Res. 2025 Jun 2;9:e66714. doi: 10.2196/66714 (PMC12171646; doi:10.2196/66714)

## **Appendix**

### **JS Go App Mockups**

1. Home Page
2. Resources Page
3. Videos Page
4. Journey Page
5. Providers Page

#### **Home page of the JS Go app**


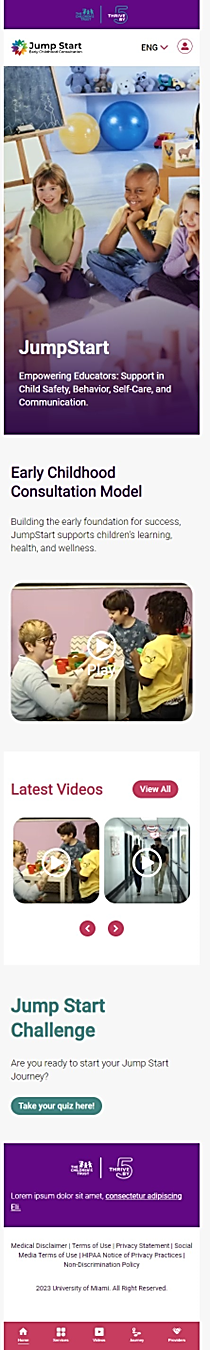


#### **Resources**


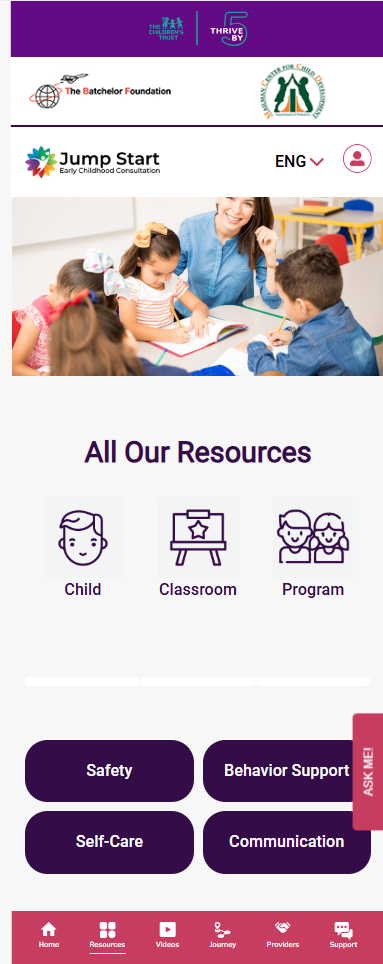


#### **Videos**


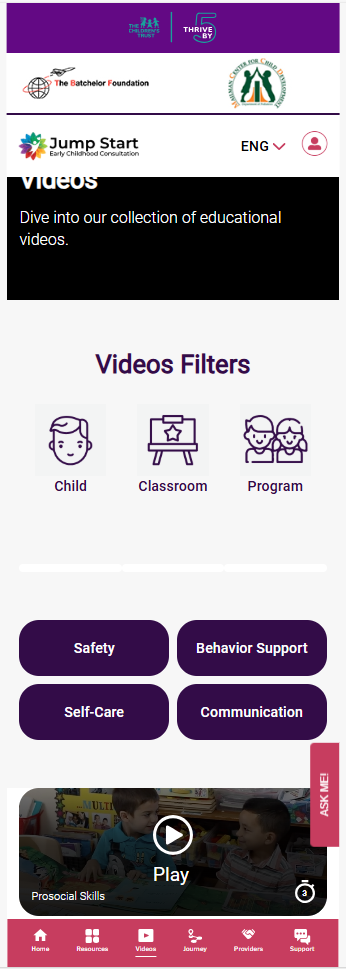


#### **Journey Page**


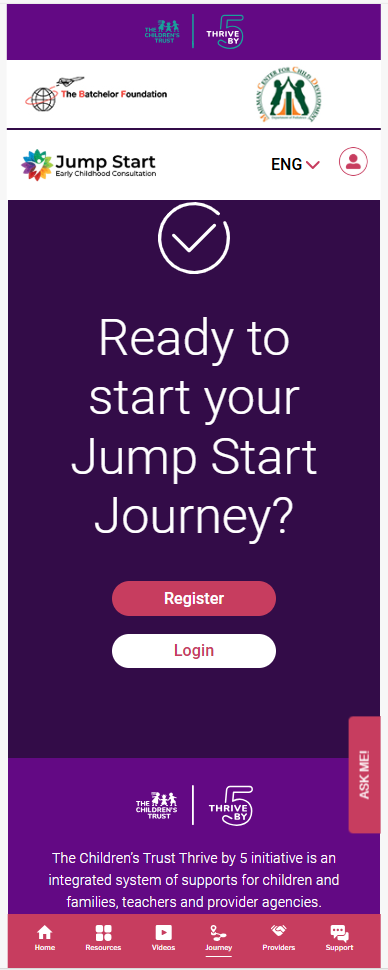


#### **Providers Page**


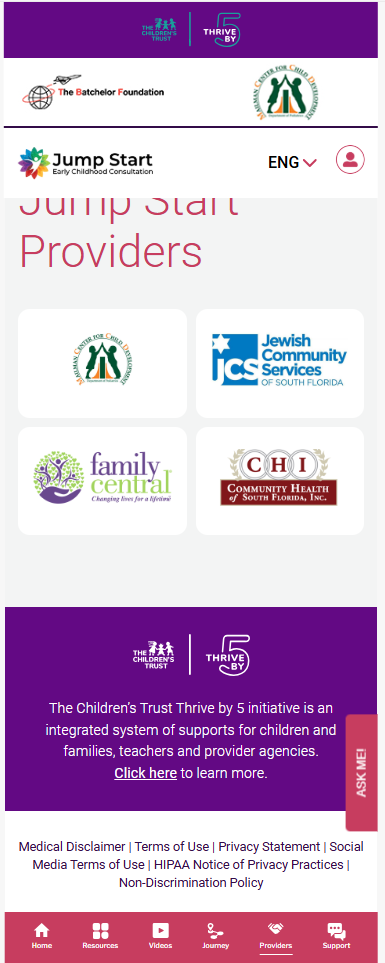

Supplement: Multimedia Appendix 1 [file formative_v9i1e66714_app1.docx]
